# Comparison of Two Approaches to Enhance Self-Esteem and Self-Acceptance in Chinese College Students: Psychoeducational Lecture vs. Group Intervention

**DOI:** 10.3389/fpsyg.2022.877737

**Published:** 2022-04-07

**Authors:** Yi Qian, Xinnian Yu, Fulian Liu

**Affiliations:** Wuxi Institute of Technology, Wuxi, China

**Keywords:** mental health, psychoeducation, self-esteem, self-acceptance, group intervention

## Abstract

**Objective:**

Self-esteem and self-acceptance are not only basic features but also influential factors of mental health. The present study aimed at assessing the effects of psychoeducational lecture and group intervention on self-esteem and self-acceptance in Chinese college students.

**Methods:**

A total of 149 Chinese college students who participated in a mental health course were randomly class-based assigned into the psychoeducational lecture group (*n* = 62) and the self-focused intervention group (*n* = 87). The lecture group received 6-session psychoeducational lectures on overview of mental health, campus adaptation, stress adjustment, self-understanding, emotion management, and interpersonal relationships. The self-focused intervention group was treated with self-related group activities involving aspects of self-knowledge, self-feeling, and self-regulation for six sessions. Pre- and post-intervention measurements were taken with Rosenberg Self-Esteem Scale and Self-Acceptance Questionnaire for both groups.

**Results:**

Self-esteem significantly increased in both groups after six sessions. However, the enhancement of self-acceptance was more robust for the self-focused intervention group than the psychoeducational lecture group.

**Conclusion:**

The psychoeducational lecture and self-focused intervention were effective approaches to improve self-esteem for Chinese college students. With respect to self-acceptance, self-focused group intervention might have a more prominent effect.

## Introduction

The college years represent a developmentally challenging transition to adulthood, during which college students are exposed to stress due to both developmental period and academic environment. Mental health has a significant influence on academic success, productivity, and social relationships in this period (Hunt and Eisenberg, 2010), because most lifetime mental disorders have the first onset by age 24 years (Kessler et al., 2005). The college mental health service should combine psychiatry, primary care, health promotion, and counseling so as to provide unique opportunities for interventions in transition-age youth that affect the life course and impact of mental illness (Downs et al., 2018).

### The Influence of Self-Esteem and Self-Acceptance on Mental Health

Self-concept could be treated as an entry point for mental health promotion in college. Numerous researches show a positive, stable, and healthy self-concept to be one of the major indicators of the development of mental health (Keltikangas-Järvinen and Räikkönen, 1990; Pillow et al., 1991; Räty et al., 2005; Grum, 2006; Ybrandt, 2008; Van Dijk et al., 2014; Busch et al., 2021). As the evaluative and affective dimension of the self-concept (Harter, 1999), the role of self-esteem in mental health was also been evidenced. Self-esteem refers to a person’s global appraisal of his/her positive or negative value, based on the scores a person gives him/herself in different roles and domains of life (Markus and Nurius, 1986; Mann et al., 2004). The vulnerability model states that low self-esteem is a risk factor for depression (Orth et al., 2016; Sakellari et al., 2020). Low self-esteem was also linked with anxiety (Du et al., 2013; Sowislo and Orth, 2013; Sakellari et al., 2020), dejection (Zeigler-Hill and Wallace, 2012), loneliness (Creemers et al., 2013; Vanhalst et al., 2013) and presence of psychological distress (Becerra et al., 2021), while high self-esteem was associated with life satisfaction (Halvorsen and Heyerdahl, 2006; Joshanloo and Afshari, 2011; Coffey and Warren, 2020) and subjective well-being (Karatzias et al., 2006; Yamaguchi et al., 2017; Yang et al., 2019). Self-esteem might be protective against psychosomatic symptoms for adolescents (ages 14–19 years; Piko et al., 2016).

Self-acceptance was the basis of self-esteem (Nathaniel, 1998), which was integrated into the multidimensional model of self-concept (Purdie and McCrindle, 2004) and was adopted to measure the level of self-concept (Green et al., 1994). Self-acceptance refers to people taking a positive attitude toward themselves and all their characteristics and reflects the degree of individual acceptance of oneself (Cai et al., 2021). Self-acceptance is crucial to mental health (Carson and Langer, 2006). Psychological well-being theory pointed out that self-acceptance was a central characteristic of positive psychological functioning and an indicator of individual psychological well-being (Ryff and Singer, 1996). A higher level of self-acceptance was associated with a lower level of anxiety, depression, negative affect, and psychological distress (Xu et al., 2016; Jibeen, 2017; Popov, 2019). A 20-year prospective cohort study found that self-acceptance decreased mortality risk by 19% and added 3 years of life even after controlling for other psychological components and potential confounders, suggesting the influence of self-acceptance on physical health as well as mental health (Ng et al., 2020). Hence, self-acceptance played a protective role in overall health development (Popov, 2019).

### The Intervention to Improve Self-Esteem and Self-Acceptance

Researchers conducted intervention studies to improve self-esteem and self-acceptance on account of their importance on mental health. High school students significantly enhanced self-esteem after entering a school-based social cognitive training group than their counterparts participating in a training group in a lecture format (Barrett et al., 1999). A four-lesson self-esteem enhancement program in school improved the self-esteem level of children in fifth and sixth grade children (Dalgas-Pelish, 2006). Researchers found that art therapy groups were effective strategies to raise the self-esteem of female juvenile offenders (Hartz and Thick, 2005). A cognitive-behavioral training program resulted in significant and long-lasting improvements in employees’ self-esteem (Proudfoot et al., 2009). The studies on college students found similar effects. College students improved self-esteem after the resistance exercise program (Moore et al., 2011), an online self-esteem and awareness training program (Sungur, 2015), and an esteem development program including practices based on music therapy, poetry therapy, and creative drama method (Yücesan and Şendurur, 2018).

With respect to self-acceptance intervention, many studies focused on special cohorts. For example, a self-acceptance group therapy based on cognitive-behavioral framework ameliorated self-acceptance, quality of life, and relevant psychological problems (e.g., depression, social anxiety) of outpatients (Schoenleber and Gratz, 2018). Forgiveness therapy increased the self-acceptance of Indonesian adult inmates (Praptomojati and Subandi, 2020). The intervention had positive effects on participants with psychological problems and even severe psychiatric conditions. For instance, the mindfulness-based stress reduction program increased the highly sensitive participants’ scores of self-acceptance, mindfulness, and emotional empathy (Soons et al., 2010). On completion of a positive psychology group therapy, participants with severe psychiatric conditions reported a significant improvement in self-acceptance and significant decreases in interpersonal sensitivity and depression (Valiente et al., 2021). Following an online intervention program, participants with elevated levels of perfectionism increased self-acceptance (Tulbure et al., 2021). However, there were few studies to explore the self-acceptance intervention effect on healthy college students.

Researchers indicated that making liberal use of psychoeducation was one of the effective strategies to reach the goal of student-centered mental health care in college setting (Downs et al., 2018). Psychoeducation could help students in less clinical distress to increase their awareness and information about mental health problems (Li et al., 2014). A brief psychoeducation course significantly improved the self-esteem levels of college students, supporting the opinion that psychoeducation was effective early intervention strategy in college (Cartwright and Hooper, 2017).

### The Present Study

As compared to western culture’s emphasis on individualism and the tendency for people in western countries to have an independent self-construal, Chinese culture places heavy emphasis on social relationships, so Chinese people’s self may not be salient as that of western people (Markus and Kitayama, 1991). However, with the substantial change of Chinese society in the past decades, self-concept and self-esteem become more and more central to youth’s development of mental health in China. Adolescents in middle school with high self-esteem experienced better mental health (Xu et al., 2020). Self-concept in Chinese college students not only influenced mental health directly, but also affected mental health indirectly through social adaptation (Zhu et al., 2016). Inconsistent with the partial mediating effect found in western and Hong Kong college students, self-concept fully mediating the role of social support on mental health was obtained in Mainland China (Xu et al., 2019). Chinese college students with high self-esteem and high self-concept reported lower interpersonal difficulties (Luo et al., 2021). Thus, researchers pointed out that Chinese students should be encouraged to evaluate their performance and worth in terms of their levels of mastery of particular tasks rather than relying on external evaluation criteria, such as social comparisons with peers (Zhou et al., 2020). It was accordingly worth enhancing self-esteem and self-acceptance of Chinese college students to prevent mental illness.

Liu et al. (2017) found that 6.24% of Chinese freshmen on average had at least one positive factor in the measurement of SCL-90 from 2005 to 2011, i.e., might have psychological problems. A recent survey study reported that more than 10% of university students in China suffered from different levels of depression, anxiety, and stress, and the mean scores of depression, anxiety, and stress all experienced downward trends during college (Gao et al., 2020). Compared to students in higher grades, freshmen’s mental health should be paid more attention to. The current study therefore particularly focused on freshmen in college and aimed to enhance their self-esteem and self-acceptance. Universities are unique places of learning where the setting has a strong focus on teaching and learning. Intervention in college improved the mental health of students through academic-based strategies (Fernandez et al., 2016). Hence, the current psychoeducational treatments were conducted within a mental health course for freshmen.

Two different psychoeducational approaches were adopted. One was a 6-session psychoeducational lecture treatment. Previous studies found lecture-based courses had positive effects on increasing mental health knowledge, hope, psychological quality, and mental health (Becker et al., 2008; Hassed et al., 2009; Shek, 2012). Through brief psychoeducation lectures about depression and knowledge of neuroscience, college students in Taiwan elevated the biological knowledge and reduced the stigma of depression (Han and Chen, 2014). However, the effects of psychoeducational lectures on self-esteem and self-acceptance have not been reported in Chinese college students.

The other psychoeducational approach was a 6-session self-focused group intervention. A prior study used group psychoeducation in which stress coping styles are taught to reduce symptoms of stress and manage the symptoms of anxiety and depression levels in Turkish nursing students (Günaydin, 2021). A paint therapy group counseling intervention significantly improved self-acceptance level in Chinese college students. The effect was still robust even in the follow-up test conducted 2 months after the intervention (Zheng et al., 2021). Nevertheless, will group intervention have similar effects on self-esteem of Chinese college students? It remained unclear.

In summary, Chinese freshmen might suffer from different levels of psychological distress. Previous studies revealed that self-esteem and self-acceptance were important indicators of mental health, which could be improved by psychoeducational treatments. Therefore, the current study adopted two psychoeducational approaches to enhance self-esteem and self-acceptance in Chinese college students, and compared the effects of psychoeducational lecture and group intervention.

## Materials and Methods

### Participants

The current study was conducted within a mandatory course “Mental Health of College Students,” which was required for all the freshmen in a higher vocational college. A total of 149 students in five classes were randomly selected from all the students in Grade 1 at class level. The participants were randomly class-based allocated into the psychoeducational lecture group (LG) or self-focused intervention group (IG). LG included 62 students from two classes majoring in Industrial Robotics and Computer Network, respectively, (57 males and 5 females, mean age 18.15 ± 0.63 years old). IG included 87 students from three classes (77 males and 10 females, mean age 18.21 ± 0.45 years). One class majored in Industrial Robotics, and the other two classes majored in Computer Network. Table 1 showed the demographic data of participants, which were not significantly different between LG and IG (*p*s > 0.05). All of the participants would get one point of the course credit after they completed the course. The study was approved by the Research Ethics Committee of Wuxi Institute of Technology.

**Table 1:** Demographic data (age, gender, and major) divided into groups.

	*n*	Age	Gender		Major	
LG	62	18.15 ± 0.63	Male	57(91.93%)	Industrial robotics	37(59.68%)
			Female	5(8.06%)	Computer network	25(40.32%)
IG	87	18.21 ± 0.45	Male	77(88.51%)	Industrial robotics	39(44.83%)
			Female	10(11.49%)	Computer network	48(55.17%)

LG, Psychoeducational lecture group; IG, Self-focused intervention group.

### Measures

#### Self-Esteem

Self-esteem was measured by the 10-item Chinese revision of the Rosenberg Self-Esteem Scale, which was widely used in China (Cai et al., 2021). Each item was rated on a scale from 1 (strongly agree) to 4 (strongly disagree). Higher scores indicated higher self-esteem. A typical item was “I feel that I’m a person of worth.” In this study, Cronbach’s *α* for self-esteem was 0.88.

#### Self-Acceptance

The Self-Acceptance Questionnaire (SAQ; Cong and Gao, 1999) was adopted to assess the degree of self-acceptance. The questionnaire is divided into two dimensions: self-acceptance (SAQ_SA) and self-evaluation (SAQ_SE). Participants responded to items on a 4-point Likert scale with values ranging from 1 (strongly disagree) to 4 (strongly agree) on the 16 items in the questionnaire. The higher the score, the higher the level of self-acceptance. A typical item was “I like my personality traits.” Internal consistencies for the current sample were Cronbach’s *α* = 0.85 for both self-acceptance and self-evaluation, and *α* = 0.88 for the whole SAQ.

#### Procedure

A pre-test/post-test study design was adopted to compare the effects of psychoeducational lecture and self-focused group intervention. Both groups completed self-esteem and self-acceptance measurements before and after the course. The instructor of the two groups was the same teacher working in the college mental health center. She has more than 10 years of experience in psychoeducation and psychological counseling.

#### Psychoeducational Lecture

Participants in the psychoeducational LG received a 6-session class-based and instructor-led psychoeducational lecture course. One session per week cost about 1.5 h. The topics of lectures in the course included: an overview of mental health, campus adaptation, stress adjustment, self-understanding, emotion management, and interpersonal relationships. The specific session content was shown in Table 2. The instructor delivered the topics in lectures with PowerPoint presentations and encouraged questions and discussion from students.

**Table 2:** Description of the psychoeducational lectures.

	Lecture topic and content
Session 1	**Overview of mental health:** introduction to the definition and standards of mental health; symptoms and treatments of psychological distress, neuroses, and mental disorder; guidelines of psychological service in college
Session 2	**Campus adaptation:** introduction to campus adaptation and maladaptation; factors and strategies of adaptation
Session 3	**Stress adjustment:** introduction to stress and its relation with mental health; Strategies for coping with stress
Session 4	**Self-understanding:** introduction and self-assessment of personality; evaluating strength and weakness; practice to accept weakness; case discussion about self-understanding
Session 5	**Emotion management:** introduction to the generation, classification, and influence of emotion; identifying, accepting, and expressing emotions; strategies for emotion regulation
Session 6	**Interpersonal relationships:** introduction to classification and factors of interpersonal relationship; principles for effective communication

#### Self-Focused Group Intervention

The 6-session class-based intervention centered on self and aimed at helping students to establish comprehensive self-knowledge, positive self-feeling, and effective self-regulation. The three aspects of self were closely linked. Each identity or self-knowledge had a particular feeling attached to it, while an individual’s behavior was determined by efforts to confirm these fundamental self-feelings (Markus and Nurius, 1986). Empirical evidence showed that gaining awareness for his/her self had a positive effect on college students’ self-esteem (Sungur, 2015), emotion regulation and self-acceptance could promote each other (Kivity et al., 2016). Accordingly, the current study adopted a self-focused group intervention involving aspects of self-knowledge, self-feeling, and self-regulation to enhance self-esteem and self-acceptance in college students. Unlike traditional didactic instruction in psychoeducational lectures, the group intervention divide the students into groups randomly and emphasized experience and expression by means of various group activities and interactions. Each session content was shown in Table 3.

**Table 3:** Description of the self-focused group intervention.

	**Session aims**	**Session activities**
Session 1	**Self-knowledge:** Get to know each other, deepen understanding and trust of group members, encourage participants to explore and understand themselves	Warm-up activities; random grouping; self-introduction in groups; making personalized self-introduction card; challenge trust circle
Session 2	**Self-knowledge:** Enhance the experience of cooperation among group members; review self-growth experience, know oneself deeply, and discover one’s uniqueness	Warm-up activity; group members standing together on a piece of paper; drawing life pictures of oneself; painting hands of oneself
Session 3	**Self-knowledge:** Discover their own strengths; learn to see themselves from different perspectives and accept themselves	Warm-up activity; evaluate oneself from different perspectives; strengths bombing; self-acceptance exercise
Session 4	**Self-feeling:** Identify one’s emotions; understand and accept one’s emotions through the connection with family of origin	Warm-up activity; my mood tree; life rainbow; a letter to inner parents
Session 5	**Self-regulation:** Clarify the goals and directions of self-growth; explore the ways to achieve them	Warm-up activity; drawing ideal life in the next 3 months; dream list; dream auction
Session 6	**Self-regulation:** Strengthen team communication and trust; improve stress coping skills; build confidence in the future	Warm-up activity; blindfolded group members lining up in a square; Chicken to Phoenix-challenge, competition, and stress; flying a dream paper plane

## Results

All the participants completed the course for six sessions (completion rate 100%). Scores in SES, SAQ, self-acceptance dimension of SAQ, and self-evaluation dimension of SAQ before and after the course for the two groups were shown in Table 4. Repeated measures ANOVA were conducted to analyze scale scores with group (LG and IG) as a between-subject factor, and time (pre- and post-) as a within-subject factor (see Table 5).

**Table 4:** The scores in pre-test and post-test for two groups.

		**LG (*n* = 62)**				**IG (*n* = 87)**			
		**Mean**	**SD**	**Min**	**Max**	**Mean**	**SD**	**Min**	**Max**
**SES score**	Pre-	28.45	4.05	15	40	30.63	4.62	22	40
	Post-	29.48	4.12	21	40	32.48	4.35	21	40
**Total score of SAQ**	Pre-	40.01	5.32	25	54	41.69	7.31	24	58
	Post-	41.80	7.30	26	64	45.40	7.04	31	64
**SAQ_SA score**	Pre-	20.4	3.49	14	30	20.95	4.42	10	29
	Post-	21.01	4.34	10	32	23.16	3.88	14	32
**SAQ_SE score**	Pre-	19.61	3.03	11	27	20.74	4.21	12	32
	Post-	20.79	4.08	14	32	22.24	4.03	15	32

LG, Psychoeducational lecture group; IG, Self-focused intervention group. SAQ_SA, Self-acceptance dimension of SAQ; SAQ_SE, Self-evaluation dimension.

**Table 5:** The intervention effects for two groups.

		***F***	***p***	**Partial *η*** ^**2**^
**SES score**	Time	19.99	0.000	0.120
	Group	16.97	0.000	0.104
	Time * Group	1.61	0.206	0.011
**Total score of SAQ**	Time	40.67	0.000	0.217
	Group	6.56	0.011	0.043
	Time * Group	4.93	0.028	0.032
**SAQ_SA score**	Time	22.13	0.000	0.131
	Group	5.072	0.026	0.033
	Time * Group	7.13	0.008	0.046
**SAQ_SE score**	Time	31.00	0.000	0.174
	Group	4.82	0.030	0.032
	Time * Group	0.43	0.513	0.003

SAQ_SA, self-acceptance dimension of SAQ; SAQ_SE, self-evaluation dimension.

**Figure 1:**
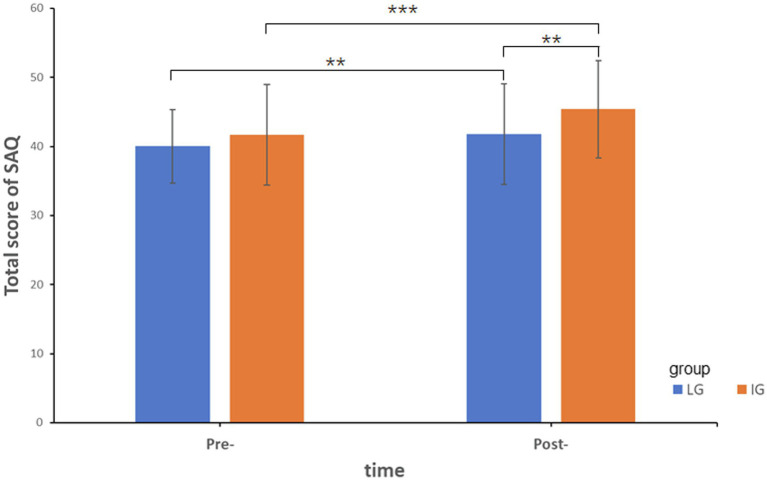
The intervention effect for total score of SAQ. ^**^p < 0.01; ^***^p < 0.001.

For SES scores, the main effects of group and time were significant [*F*(1,147) = 16.97, *p* < 0.001, partial *η*^2^ = 0.104; *F*(1,147) = 19.99, *p* < 0.001, partial *η*^2^ = 0.12], suggesting that IG had higher SES scores than LG while post-SES score was significantly higher than pre-test. However, the interaction between group and time was not significant. With respect to the scores in the full SAQ, the main effects of group and time were significant [*F*(1,147) = 6.56, *p* < 0.05, partial *η*^2^ = 0.043; *F*(1,147) = 40.66, *p* < 0.001, partial *η*^2^ = 0.217], the interaction between group and time was also significant [*F*(1,147) = 4.93, *p* < 0.05, partial *η*^2^ = 0.032]. Further simple effect analysis (see Figure 1) showed that post-score in SAQ were significantly higher than pre-score for both groups [LG: *F*(1,147) = 10.38, *p* < 0.01, partial *η*^2^ = 0.066; IG: *F*(1,147) = 31.65, *p* < 0.001, partial *η*^2^ = 0.177]. There was no significant difference between the two groups in the pre-test, while IG had a significantly higher score than LG in the post-test [*F*(1,147) = 9.06, *p* < 0.01, partial *η*^2^ = 0.058].

**Figure 2:**
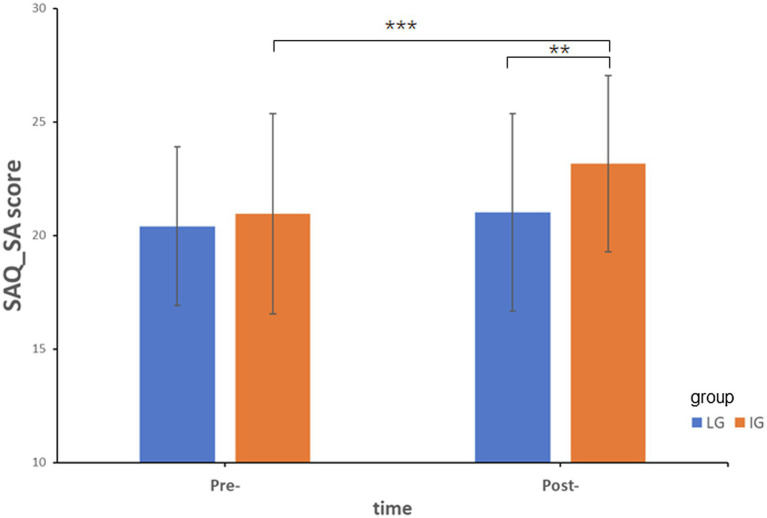
The intervention effect for the score of self-acceptance dimension in SAQ. ^**^p < 0.01; ^***^p < 0.001.

In order to distinguish the change of scores in either dimension of SAQ, ANOVA was conducted, respectively. For self-acceptance dimension, the main effects of group and time were significant [*F*(1,147) = 5.07, *p* < 0.05, partial *η*^2^ = 0.033; *F*(1,147) = 22.13, *p* < 0.001, partial *η*^2^ = 0.131], the interaction between group and time was also significant [*F*(1,147) = 7.13, *p* < 0.01, partial *η*^2^ = 0.046]. Simple effect analysis (see Figure 2) showed post-score in self-acceptance dimension was significantly higher than pre-score for IG [*F*(1,147) = 23.29, *p* < 0.001, partial *η*^2^ = 0.137], while there was no difference for LG. There was no significant difference between the two groups in the pre-test, while IG had a significantly higher score than LG [*F*(1,147) = 9.69, *p* < 0.01, partial *η*^2^ = 0.062] in the post-test. For self-evaluation dimension, there were significant main effects for group and time [*F*(1,147) = 4.82, *p* < 0.05, partial *η*^2^ = 0.032; *F*(1,148) = 31.00, *p* < 0.001, partial *η*^2^ = 0.174]. Participants in IG had higher scores in the self-evaluation dimension. The post-score was significantly higher than the pre-score.

## Discussion

The present study compared the influences of psychoeducational lecture and group intervention on self-esteem and self-acceptance in Chinese college students. The results indicated that participants significantly improved self-esteem scores after course in both groups, suggesting both psychoeducational approaches were effective for self-esteem enhancement. According to the higher scores in the post-SAQ of IG than that of LG, it was implied that self-focused group intervention was more effective for self-acceptance enhancement than lecture. In further analysis distinguishing dimensions of SAQ, we found a more obvious advantage of group intervention than psychoeducational lectures in enhancing self-acceptance, while a comparable effect of both approaches on self-evaluation.

The psychoeducational approaches were effective in increasing self-esteem no matter whether it was lecture-based or activity-based. This finding was consistent with previous studies, which obtained significant enhancement of self-esteem by means of psychoeducational lectures (Cartwright and Hooper, 2017), art therapy (Yücesan and Şendurur, 2018), or online training (Sungur, 2015). In addition, the present study found that the level of self-evaluation dimension of SAQ was also improved regardless of psychoeducational approaches. Self-esteem was regarded as the evaluative dimension of the self-concept and referred to total evaluations towards oneself (Harter, 1999). In the present study, the items in the self-evaluation dimension of SAQ resembled those in SES. For example, “I have many strengths and few faults.” (the second item in SAQ) and “I feel that I have a number of good qualities” (the second item in SES) had similar meanings. The close association between self-evaluation and self-esteem might lead to the same effect pattern.

The treatment of psychoeducational lecture played a role in enhancing self-esteem and self-evaluation, which might be related to the reduce of stigma. The lectures in the present study delivered information about causes, factors, and coping strategies of psychological distress and mental illness. Han and Chen (2014) indicated that a 30-min neurobiology-based psychoeducational lecture significantly reduced the social distance from depressed individuals. A meta-analysis study supported the positive effects of psychoeducation on reducing stigma for mental illness (Corrigan et al., 2012). Meanwhile, self-stigma of mental illness and self-stigma of seeking psychological help predicted decreased self-esteem (Lannin et al., 2015). It was accordingly speculated that the current psychoeducational lectures might reduce stigma for psychological distress, and further increase self-esteem and self-evaluation of college students.

Consistent with an online self-esteem and awareness training study (Sungur, 2015), self-focused group intervention in the present study improved self-esteem and self-evaluation of college students. The intervention highlighted the awareness of self-knowledge, self-feeling, and self-regulation. Individuals who were self-aware gained insight into their feelings and thoughts, instead of focusing their attention on their negative aspects, accepted their positive and negative sides. Thus, they were open to criticism and increased their tolerance to adverse experiences (Sungur, 2015). Self-regulation facilitated identity achievement which further increased self-esteem (Hofer et al., 2011). Increasing self-awareness was beneficial to identifying personal preferences, values, and life purpose and creating a realistic appreciation of personal strengths and weaknesses (Hartz and Thick, 2005). Students were encouraged to set realistic goals and take steps to achieve them, and finally improved self-esteem.

In comparison with psychoeducational lectures, self-focused group intervention had a more robust effect on the improvement of self-acceptance. There were similarities between self-acceptance and self-esteem, but there were also differences between the two concepts (Macinnes, 2006). Self-acceptance was tapping into a general level of psychological well-being while self-esteem is more associated with the issues surrounding the development and maintenance of depressive thoughts (Macinnes, 2006). Popov (2019) found self-acceptance was a better predictor of mental health than self-esteem. These previous findings suggested self-acceptance influenced mental health in a more fundamental and extensive way. Combined with the results in the present study, it seemed harder to improve self-acceptance than self-esteem. Self-acceptance enhancement might rely more on interpersonal interaction rather than didactic psychoeducation. Interaction experienced within the group helped students to share all their emotions and thoughts freely and confidently. This experience brought along the acquisition of the skill to listen and understand first themselves, then each other without judging (Yücesan and Şendurur, 2018). In-group interaction and sharing processes might set the basis for the development of self-acceptance.

In summary, the present study explored the effects of psychoeducational approaches on self-esteem and self-acceptance in college setting. The results manifested both psychoeducational lecture and self-focused group intervention significantly improved self-esteem for Chinese college students. However, self-acceptance increased more obviously after group intervention. The findings might provide insights for mental health promotion for college students. Nonetheless, there were some limitations. Firstly, the two groups were not equivalently matched due to that sample selection was randomly class-based among all the freshmen. Participants in IG had higher scores in pre-SES measurement than LG. ANCOVA with the pre-score in SES as covariate showed that IG had significantly higher post-score in SES than LG [*F*(1,146) = 8.88, *p* < 0.01, partial *η*^2^ = 0.057] after controlling the baseline difference, but the better effect of group intervention on self-esteem should be further examined by strictly matching baselines. Secondly, the current study lacked measurements directly towards mental health, so it was unclear whether the enhancement of self-esteem and self-acceptance could be transferred to general mental health. Thirdly, follow-up measurement was not conducted in months after intervention, so the sustainability of intervention effect remained unclear. Fourthly, the current sample was drawn from one college in China and was insufficient to represent all the Chinese college students. Future studies should recruit larger samples from colleges in different areas and different levels in China to examine the intervention effects. Due to the limitations, caution was needed in drawing conclusions of psychoeducation effects. Further studies were required to confirm the current conclusion by strictly matching participants, enlarging sample size, adding mental health measurements and follow-up tests.

## Data Availability Statement

The original contributions presented in the study are included in the article/supplementary material, further inquiries can be directed to the corresponding author.

## Ethics Statement

The studies involving human participants were reviewed and approved by Wuxi Institute of Technology. The patients/participants provided their written informed consent to participate in this study.

## Author Contributions

YQ designed the study, analyzed the data, and wrote the manuscript. XY collected the data. FL helped to design the study and collect the data. All authors read and approved the final manuscript.

## Funding

This research was supported by the grants from Humanity and Social Science Youth Foundation of Ministry of Education of China (17YJC190021), the Natural Science Foundation of Jiangsu Province (BK20170180), and Doctoral research project of Wuxi Institute of Technology (BS201904).

## Conflict of Interest

The authors declare that the research was conducted in the absence of any commercial or financial relationships that could be construed as a potential conflict of interest.

## Publisher’s Note

All claims expressed in this article are solely those of the authors and do not necessarily represent those of their affiliated organizations, or those of the publisher, the editors and the reviewers. Any product that may be evaluated in this article, or claim that may be made by its manufacturer, is not guaranteed or endorsed by the publisher.
